# Cancer support in the era of virtual interaction: analysis of a community-based cancer support organization

**DOI:** 10.1007/s00520-025-10110-x

**Published:** 2025-11-06

**Authors:** Carley L. Mitchell, Jeffrey Zhong, Qian Wang, Melinda L. Hsu

**Affiliations:** 1 Department of Medicine, University Hospitals Cleveland Medical Center, Seidman Cancer Center, Cleveland, OH USA; 2 Case Western Reserve University School of Medicine, Cleveland, OH USA

**Keywords:** Supportive oncology, Telehealth, Disparities, Community, Caregiver

## Abstract

**Purpose:**

Several benefits exist when supportive therapies (exercise, art, mind-body practices, support groups, etc.) are incorporated into care for those with cancer. Research assessing real-world disparities among support service utilization is limited, especially as virtual options have increased. We aimed to identify changes in participant demographics and resource utilization at a community-based cancer support organization as program delivery shifted from all in-person to virtual and hybrid platforms.

**Methods:**

Deidentified, retrospective data was gathered from three 6-month time periods, corresponding to in-person, virtual, and hybrid program delivery. Participant demographics, cancer diagnoses, and types of programs attended were collected. Comparisons between individual variables were performed in relation to the distinct 6-month time periods. The Wilcoxon test was used for age, and chi-square tests were used for all other variables. Statistical significance was set at two-sided *p*-values < 0.05.

**Results:**

Participants of female gender, white race, and above-average household income defined the majority and did not change based on delivery platform. However, participants utilizing services in the virtual/hybrid setting were significantly younger with fewer breast cancer diagnoses compared to in-person delivery. Engagement in support, exercise, art, and mind/body programs significantly decreased while educational participation significantly increased from in-person to virtual/hybrid settings.

**Conclusions:**

Participation was highly skewed toward female, white, and higher-income individuals. Younger participant age in the virtual setting mirrors the age-related disparity among telemedicine utilization. While virtual participation led to decreased engagement in programs emphasizing camaraderie and social bonding, participation in programs encompassing more passive engagement remained steady or rose. This study identifies important gaps in support service utilization and underscores the importance of in-person interaction.

## Introduction

Cancer care delivery is largely multifaceted and cannot be fully successful with a narrow team approach. A variety of supportive therapies have demonstrated significant benefits to patients with cancer when used in combination with traditional medical approaches. Symptoms such as fatigue, lymphedema, anxiety, depression, and declining functional ability can be mitigated through tailored exercise programs [1]. Anxiety, depression, and pain can improve via massage, acupuncture, and mind-body practices [2, 3]. Furthermore, individual therapy and support groups provide safe mediums of self-expression and allow for community development among those with shared experiences. Individuals participating in these groups often develop improved coping mechanisms, have decreased rates of mental health conditions, and increased quality of life [4].

Fortunately, many national non-profits, hospital- and community-based organizations exist providing access to the above services. Ultimately, with a large focus on the psychosocial aspect of suffering, these programs help create a full comprehensive care experience that is difficult to replicate in the clinic setting [5]. The Gathering Place (TGP) is one such community-run, non-profit organization serving the Greater Cleveland area. With a mission to support, educate, and empower individuals and families currently coping with the impact of cancer, they have engaged more than 47,000 individuals since the year 2000 [6]. Multiple programs and services are offered to participants, free of charge, including nutrition and fitness programs, art and music therapy, individual and group support services, mind/body practices, and educational engagement.

Over the past few years, there has been a major shift in healthcare delivery through the adoption of telehealth platforms largely resulting from the COVID-19 pandemic. This change was not only isolated to medical visits but expanded to include the broader array of support services offered by organizations like TGP. Prior to 2020, all services at TGP were offered in-person (IP). However, in 2021, during the height of the pandemic, all services shifted to a virtual (V) setting, which then evolved into a hybrid (H) approach in 2022. While racial/ethnic, socioeconomic, and age-related disparities exist among telehealth utilization within the cancer population [7, 8], it is unclear if similar variations exist among support group and wellness program participation.

Our study aimed to uncover basic demographic and cancer-specific data among participants at TGP. Further, we aimed to examine the changes in participant demographics and program utilization throughout the shift from IP to V to H program delivery platforms.

## Methods

Retrospective participant data was gathered from TGP’s internal database. While participants may have been referred via their healthcare provider, a referral is not required, and engagement may be initiated through word of mouth. Three 6-month periods were queried based on evolving program delivery platforms. Date ranges for respective IP, V, and H delivery platforms included 01/01/2019–06/30/2019, 01/01/2021–06/30/2021, and 01/01/2022–06/30/2022. Collected data included participant type, age, sex, race, residential zip code, cancer type, and programs utilized. Participant type was divided into two categories—those personally diagnosed with cancer (referred to as participants with cancer) and those indirectly affected by cancer, including family or friends (referred to as caregivers). Residential zip codes were used to approximate median household income via the U.S. Census Bureau [9]. Cancer diagnoses were categorized as breast, gynecologic, hematologic, lung, prostate, gastrointestinal, pancreatic, genitourinary, central nervous system, head/neck, bone/soft tissue, melanoma, liver/biliary tract, or unknown. Individual program offerings were categorized as support, education, exercise, nutrition, art or mind/body, or other. All data analyzed in this observational study was fully de-identified and collected by TGP as part of their standard practice. No interaction or intervention occurred between the research team and TGP participants. As such, IRB approval was not required.

### Statistical analysis

Participant demographics and program data were compared among IP, V, and H platforms using the Wilcoxon test for continuous variables and chi-square tests for categorical variables, stratified by participant type. The analysis was conducted using SAS 9.4 (SAS Institute, Cary, NC). The significance level was set at a two-sided *p* < 0.05.

## Results

### Participant demographics

For simplicity, basic participant demographics are reported using the 2022 cohort. This is considered most likely present conditions due to the hybrid program offering, which is actively utilized by TGP today. During this 6-month time frame, there were 1029 participants diagnosed with cancer who had a median age of 62 years. Most participants were women (84.6%) and of white race (76.2%) with a projected median household income of $69,965. Participants with breast cancer (33.6%) far outnumbered others, followed by gynecologic (7.0%) and hematologic malignancies (6.2%) (Table 1).

**Table 1:** Demographics of participants with cancer by program platform/year (*n* = 2974)

	In-person (2019) (*n* = 1221)	Virtual (2021) (*n* = 724)	Hybrid (2022) (*n* = 1029)	*p*-value
Age (median, IQR)	77.5 (75–88)	62 (51–70)	62 (52–70)	< 0.0001
Sex, *n* (%)				0.55
Female	1016 (83.2)	599 (82.7)	870 (84.6)	
Male	205 (16.8)	125 (17.3)	159 (15.5)	
Race, *n* (%)				0.19
Caucasian	897 (73.5)	560 (77.4)	784 (76.2)	
African American	196 (16.1)	99 (13.7)	161 (15.7)	
Other	128 (10.5)	65 (9.0)	84 (8.2)	
Income (median, IQR)	69,965 (57,634–91,598)	69,965 (57,634–91,589)	69,965 (51,266–91,598)	0.66
Cancer type, *n* (%)				< 0.0001
Breast	578 (47.3)	376 (36.5)	243 (33.6)	
GYN	108 (8.9)	107 (10.4)	51 (7.0)	
Hematological	116 (9.5)	78 (7.6)	45 (6.2)	
Lung	66 (5.4)	77 (7.5)	39 (5.4)	
Prostate	85 (7.0)	37 (3.6)	27 (3.7)	
GI	71 (5.8)	54 (5.3)	20 (2.8)	
Pancreas	35 (2.9)	27 (2.6)	25 (3.5)	
GU	35 (2.9)	20 (1.9)	10 (1.4)	
CNS	35 (2.9)	19 (1.9)	10 (1.4)	
Head/neck	38 (3.1)	15 (1.5)	5 (0.7)	
Bone/soft tissue	17 (1.4)	17 (1.7)	12 (1.7)	
Melanoma	13 (1.1)	12 (1.2)	15 (2.1)	
Liver/biliary tract	6 (0.5)	12 (1.2)	10 (1.4)	
Unknown	18 (1.5)	178 (17.3)	212 (29.3)	

Similar demographics were observed among caregiver participants. During the same period, there were 343 caregiver participants with a median age of 54 years. 77% were women and 80.2% were of white race with a projected median household income of $74,569. Participants impacted by breast cancer (11.7%), hematologic malignancies (11.7%), and gastrointestinal cancers (11.1%) were of similar frequencies. One third of caregiver participants attended TGP following the loss of a loved one (Table 2).

**Table 2:** Demographics of caregiver participants by program platform/year (*n* = 1339)

	In-person (2019) (*n* = 711)	Virtual (2021) (*n* = 285)	Hybrid (2022) (*n* = 343)	*p*-value
Age (median, IQR)	58 (41–70)	47 (31–65)	54 (39–67)	< 0.0001
Sex, *n* (%)				0.11
Female	512 (72.0)	220 (77.2)	264 (77.0)	
Male	199 (28.0)	65 (22.8)	79 (23.0)	
Race, *n* (%)				0.37
Caucasian	531 (74.7)	214 (75.1)	275 (80.2)	
African American	77 (10.8)	30 (10.5)	31 (9.0)	
Other	103 (14.5)	41 (14.4)	37 (10.8)	
Income (median, IQR)	74,569 (58,714–91,598)	73,953 (64,766–94,072)	74,569 (59,321–91,598)	0.35
Cancer type, *n* (%)				< 0.0001
Breast	176 (24.8)	64 (22.5)	40 (11.7)	
GYN	52 (7.3)	14 (4.9)	19 (5.5)	
Hematological	77 (10.8)	28 (9.8)	40 (11.7)	
Lung	89 (12.5)	19 (6.7)	31 (9.0)	
Prostate	46 (6.5)	18 (6.3)	18 (5.3)	
GI	46 (6.5)	23 (8.1)	38 (11.1)	
Pancreas	51 (7.2)	20 (7.0)	30 (8.8)	
GU	28 (3.9)	12 (4.2)	16 (4.7)	
CNS	56 (7.9)	16 (5.6)	14 (4.1)	
Head/neck	30 (4.2)	3 (1.1)	12 (3.5)	
Bone/soft tissue	16 (2.3)	18 (6.3)	16 (4.7)	
Melanoma	9 (1.3)	11 (3.9)	9 (2.6)	
Liver/biliary tract	21 (3.0)	8 (2.8)	8 (2.3)	
Unknown	14 (2.0)	31 (10.9)	52 (15.2)	
Grief/loss				0.01
Yes	185 (26.0)	98 (34.4)	110 (32.1)	
No	526 (74.0)	187 (65.6)	122 (67.9)	

### Demographic changes based on program platform

During the progression from IP to V to H program delivery, the main shift in participant demographics was age. The median age of participants with cancer decreased from 77.5 (IP) to 62 years (V and H) (*p* < 0.0001) (Table 1). Similarly, caregiver participant median age decreased from 58 (IP) to 47 years (V), followed by an increase to 54 years during the H period (*p* < 0.0001) (Table 2). Cancer type also significantly changed among both participants with cancer and caregivers. Most notably, breast cancer diagnoses appeared to decrease over time, while rates of unknown cancer diagnoses increased (*p* < 0.0001). Other demographics including sex, race, and projected household income did not significantly differ as program platforms changed.

### Program participation based on program platform

TGP offers numerous programs, falling under larger domains including support, education, exercise, nutrition, and art or mind/body. The percentage of participants with cancer involved in support (27.8% vs 22.3%, *p* < 0.0001 [IP vs H]; 25.8% vs 22.3%, *p* = 0.01 [V vs H]), exercise (18.7% vs 16.3%, *p* = 0.03 [IP vs V]; 18.7% vs 12.7% *p* < 0.0001 [IP vs H]; 16.3% vs 12.7% *p* < 0.01 [V vs H]), and art or mind/body (10.6% vs 6.2%, *p* < 0.0001 [IP vs V]; 10.6% vs 4.9%, *p* < 0.0001 [IP vs H]) programs decreased as the platform shifted from IP to V to H, whereas the percentage involved in education (19.5% vs 34.6%, *p* < 0.0001 [IP vs V]; 19.5% vs 37.2%, *p* < 0.0001 [IP vs H]) and nutrition (11.4% vs 13.1%, *p* = 0.04 [IP vs H]) programs increased.

Similar results were identified among caregiver participants. The percentage of caregivers engaged in support (42.2% vs 33.9%, *p* < 0.0001 [IP vs V]; 42.2% vs 33.0% *p* < 0.0001 [IP vs H]), exercise (10.9% vs 6.6%, *p* < 0.001 [IP vs V]; 10.9% vs 6.7%, *p* < 0.001 [IP vs H]) and art or mind/body (6.8% vs 3.9%, *p* < 0.01 [IP vs V]; 6.8% vs 4.4%, *p* = 0.02 [IP vs H]) programs decreased during the platform transition from IP to V to H, whereas the percentage of caregivers engaged in education (22.3% vs 30.1%, *p* < 0.0001 [IP vs V]; 22.3% vs 32.4%, *p* < 0.0001 [IP vs H]) programs increased.

**Fig. 1:**
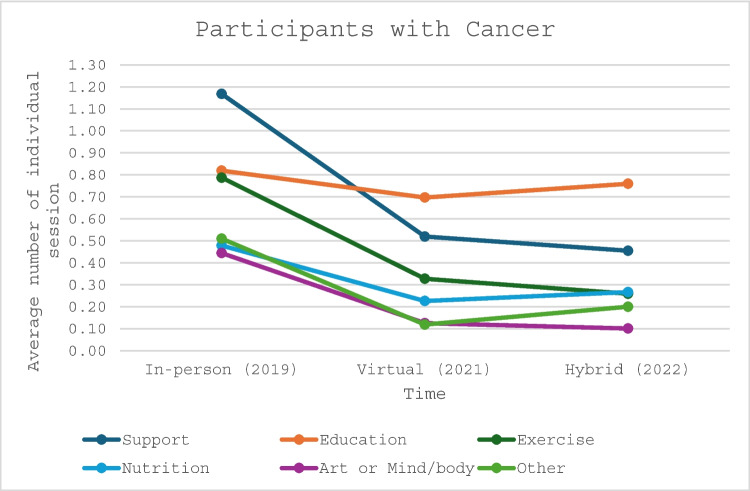
Average number of individual sessions attended by participants with cancer

**Fig. 2:**
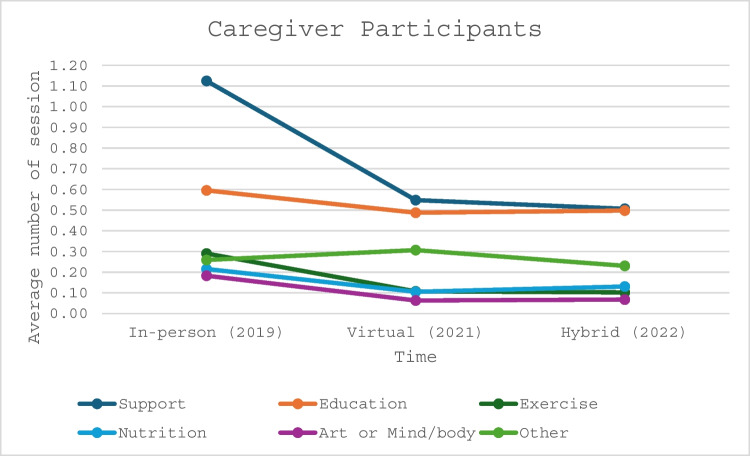
Average number of individual sessions attended by caregiver participants

Additionally, the average number of individual sessions each participant with cancer and caregiver participant engaged in decreased from IP to V and H delivery (Figs. 1 and 2). There was a significant decline among all program categories for participants with cancer when comparing IP to V and H delivery platforms. The average total sessions dropped from 4.21 to 2.10 and 2.04 (*p* < 0.0001 and *p* < 0.0001) (Table 3), respectively. Similar findings were recognized among caregiver participants with average total sessions declining from 2.67 to 1.62 and 1.53 (*p* < 0.0001 and *p* < 0.0001), respectively (Table 4).

**Table 3:** Comparing the average number of individual sessions per each participant with cancer

	In-person (2019)	Virtual (2021)	Hybrid (2022)	*p*-value 2019 vs 2021	*p*-value 2019 vs 2022	*p*-value 2021 vs 2022
Support	1.17	0.52	0.45	< 0.0001	< 0.0001	0.0037
Education	0.82	0.70	0.76	< 0.0001	0.0003	0.0026
Exercise	0.79	0.33	0.26	< 0.0001	< 0.0001	0.0014
Nutrition	0.48	0.23	0.27	< 0.0001	< 0.0001	0.0480
Art or mind/body	0.44	0.13	0.10	< 0.0001	< 0.0001	0.1007
Other	0.51	0.12	0.20	< 0.0001	< 0.0001	< 0.0001
Total	4.21	2.01	2.04	< 0.0001	< 0.0001	0.6901

**Table 4:** Comparing the average number of individual sessions per each caregiver

	In-person (2019)	Virtual (2021)	Hybrid (2022)	*p*-value 2019 vs 2021	*p*-value 2019 vs 2022	*p*-value 2021 vs 2022
Support	1.12	0.55	0.51	< 0.0001	< 0.0001	0.2004
Education	0.60	0.49	0.50	0.0001	0.0007	0.7496
Exercise	0.29	0.11	0.10	< 0.0001	< 0.0001	0.8040
Nutrition	0.22	0.11	0.13	< 0.0001	0.0002	0.2278
Art or Mind/body	0.18	0.06	0.07	< 0.0001	< 0.0001	0.7867
Other	0.26	0.31	0.23	0.0611	0.2530	0.0085
Total	2.67	1.62	1.53	< 0.0001	< 0.0001	0.1836

## Discussion

Overall, this study demonstrates several findings. First, we identified that participation at TGP is most pronounced among females, the white racial majority, and those of higher socioeconomic status (SES). Additionally, female-prone malignancies such as breast and gynecologic cancers accounted for at least 40% of overall diagnoses during each period analyzed, and finally, there were significant changes in program participation associated with the transition to virtual program delivery.

### Demographic disparities

To adequately understand demographic disparities among participants at TGP, it is important to recognize the baseline demographic data of Cuyahoga County, where TGP offers services. In 2022, the sex and racial demographics of Cuyahoga County reveal a slight female predominance, with 63.1% of all individuals identified as white and 30.5% identified as black [9]. Per the Ohio Department of Health in 2021, Cuyahoga County’s cancer incidence was higher among males compared to females (525.8/100,000 vs 450.1/100,000 people), while cancer incidence was similar among white and black individuals (475.9/100,000 vs 473.1/100,000 people) [10].

Despite an increased cancer incidence among males in the region, female participation at TGP surpassed that of males. This gender distribution is comparable to other reports [11–15]. Compared to women, who often seek emotional support and camaraderie during a cancer diagnosis, men are reported to have a greater interest in gaining information regarding their cancer [12, 16], which is widely achievable through internet-based searches [17]. These differences are likely influenced by social constructs of masculinity, augmenting male coping behavior compared to females [17–19]. For example, a meta-analysis describing coping methods among men diagnosed with prostate cancer identified two themes associated with the masculine identity: avoidance, minimization, and withdrawal, along with retaining pre-illness identity and lifestyle. The first embodies disengagement with the diagnosis physically, psychologically, and interpersonally, while the latter attempts to maintain normal gender roles [18]. Another study identifies similar sentiments such as distress with changing roles from provider or protector to one who requires care [19]. Ultimately, the theme reframing masculinity and seeking support, which involves the deconstruction of the masculine identity, allowing for greater vulnerability, accepting weakness, expressing emotion, and support seeking, is considered a highly adaptive process [18]. It could be argued that a minority of men develop this coping strategy, contributing to the gender disparities seen among many psychosocial support services. The comparatively low rates of male patients with cancer utilizing support systems such as TGP highlight the specific need to develop effective support strategies tailored to this population. Additionally, studies suggest that men rely heavily on their partners or spouses for psychosocial support throughout a cancer diagnosis [17, 19], so determining optimal ways to support these caregivers is also of great importance.

Further, we found that racial demographics are highly skewed toward white individuals compared to those of black race when considering the baseline population data of Cuyahoga County. Once again, this result is in line with prior reports suggesting support groups and services are more frequently attended by those of white race [11]. Multiple factors may limit black individuals from seeking such support including a propensity for cultural silence surrounding cancer diagnoses or feeling unable to relate to peers as a racial minority among predominantly white attendees [20]. Additionally, seeking support through faith-based practice has been cited as one of the most important layers of support for black women diagnosed with breast cancer [20]. Notably, TGP is not religiously affiliated. Further, some racial minorities may exhibit decreased awareness of available support groups/services and may require encouragement from family and healthcare providers prior to successful integration [21]. To ensure organizations like TGP are inclusive and provide equal benefits to those who may be underserved, it is imperative to focus on culturally competent integration of support services. This may be accomplished through incorporation of a racially diverse and/or multi-lingual support staff, offering race-specific support groups, etc. Healthcare providers must also engage patients of racial minorities to determine their level of support needs and offer and encourage participation in resources that are available.

Finally, most studies have reported increased support group participation among younger individuals [12, 13, 15, 22], which is consistent with results from our analysis. Median cancer participant age was slightly lower, at 62 years, compared to 66 years, the median age of all cancer diagnoses. The caregiver participant’s median age was even younger at 54 years. Additionally, we found a higher projected median household income for participants with cancer and caregivers, at $69,965 and $74,569, respectively, compared to the 2021 median household income of $55,109 among those residing within Cuyahoga County [9]. Mixed data has been reported regarding SES and those engaged in support services. Some studies have identified a participation skew toward middle-income and higher SES [15, 22], while some have not revealed any apparent shift [11]. One practical explanation for this finding in our study may be related to the physical location of TGP, with both branches situated in more affluent communities. Many reports have indicated that cancer survivors of low SES suffer from poorer health-related quality of life [23]. Therefore, it is important to prioritize making these, often no-cost, services accessible to those of all financial backgrounds. This could be accomplished by expanding services directly into underserved areas or offering participation options at outpatient medical facilities where patients are frequently present for medical follow-up appointments.

### Cancer-related disparities

Consistent with the larger US population, breast (14.9%), lung (13.9%), and prostate (12.7%) cancers account for the highest incidence rates in Cuyahoga County [10], while lung (25.6%), colon/rectum (8.4%), and breast (7.7%) cancers represent the highest mortality rates [10]. However, diagnoses among participants with cancer at TGP did not mirror these distributions. Referring to the 2022 data, breast cancer diagnoses far outweighed others at 33.6% of participants, while lung and prostate cancer diagnoses were much lower at 5.4% and 3.7%, respectively. Importantly, gynecologic cancer diagnoses were the second leading cancer type, correlating with the skew toward female participation. On the other hand, caregiver participants were most frequently impacted by breast cancer (11.7%), hematologic malignancies (11.7%), and gastrointestinal cancers (11.1%).

While a study from the United Kingdom reported prostate cancer as the second leading diagnosis among support group participants, preceded by breast cancer and followed by hematologic malignancies [11], Grand et al. showed a comparable distribution to our current study with breast, hematologic, and female genital cancers comprising the top three diagnoses [13]. Low participation rates among individuals diagnosed with prostate cancer may be related to factors associated with male sex, as reported above. One consistent finding is limited involvement among patients diagnosed with lung cancer. This is notable given the high incidence and mortality rate corresponding to this diagnosis. Based on a study looking at the preferred support group format among lung cancer patients, they identified preferences for a singular disease focus, a setting located within the medical facility, and facilitation by a healthcare provider [24]. Patients with lung cancer may have increased concerns regarding the stigma of their diagnosis, poor prognosis, and minimal advocacy work compared to other diagnoses like breast and prostate cancers [25]. And similar to male participants, they often place a greater emphasis on information sharing and self-care as opposed to emotional/psychological support [24]. It is evident that specific needs can vary between individual malignancies. Each may produce unique side effects and stressors, making generalized support group strategies difficult. Further exploration into these variations is warranted.

Ultimately, the demographics revealed from the 2022 TGP data are grossly in line with many other studies reporting on the utilization of support groups among those with cancer. Our results add to this repository, but also provide needed insight into the demographic composition of an easily accessible organization providing a broader array of services that includes but is not limited to support. Many organizations similar to TGP exist; however, reports suggesting their reach are not well defined in the literature.

### Demographics based on delivery platform

As the program delivery platform shifted, the median age among participants diagnosed with cancer significantly decreased from IP to V and H settings—77.5 years to 62 years. Additionally, cancer type also revealed a significant shift; most notably, a decrease in breast cancer diagnoses. However, it should be recognized that unknown cancer diagnoses increased during this time from 1.5% (IP) to 17.3% (V) to 29.3% (H), making the interpretation of these results challenging. Similar demographic changes were noted among caregiver participants, as well.

Previous studies have demonstrated that those of older age, racial minority, living in rural areas, and having lower SES led to poorer telehealth uptake among respective cancer patients [7, 8]. While our study only revealed changes in age favoring younger participants as the virtual platform rose, race and projected household income remained consistent. However, this is in the setting of a baseline skew toward the white, racial majority, and above-average household income.

### Program participation based on delivery platform

During the timeframe where TGP operated solely on an IP basis, we found that 27.8% of participants with cancer attended at least one support program (i.e., a support group or one-on-one support session). During this time, support was the most frequently utilized program category. However, as the option for virtual attendance was established, the percent of participants engaging in support programs significantly decreased to 22.3% in the H setting. A similar trend was observed among exercise and art or mind/body programs. Interestingly, the opposite trend developed among education and nutrition programs. When offered only on an IP basis, 19.5% of participants engaged in education, while 37.2% of participants utilized education programs when given the option for virtual attendance. Similar results were seen among caregiver participants.

Online support participation is more frequently sought out and utilized by younger individuals [26, 27]. While several advantages of online support include increased availability, anonymity, lower commitment, etc., online support may be better suited for information sharing compared to emotional support [28]. Interestingly, virtual platforms have been shown to promote easier self-expression, possibly related to relative anonymity, but face-to-face interactions foster closer relationships leading to greater group involvement, personal recognition, emotional support, giving and receiving care, etc. [26, 29]. It is possible that the declining support group participation following the adoption of the virtual platform by TGP was due to a decreased sense of camaraderie among participants, especially for those who were used to face-to-face interactions. Arguably, exercise and art or mind/body programs also benefit from physical connection and group camaraderie, making this difficult to achieve in virtual settings. This may have also contributed to their apparent decline during V and H periods. Finally, nutrition and, to a greater extent, education programs could be considered more passive group opportunities that may be more easily facilitated in the virtual setting and provide similar benefits when compared to in-person delivery. This may have accounted for their increased uptake among participants during V and H periods.

## Limitations

Our study has several limitations. Due to its descriptive methodology, we are unable to determine direct causes for observed results, and prospective analyses are warranted to explore causality. Further, the internal database utilized by TGP was not consistent throughout the three time periods analyzed, and mandatory reporting among variables was not uniform. Therefore, varying levels of unknown data were identified, which could impact results from this study. Finally, it is unclear how the frequency or quantity of available programs among each category changed throughout the study periods. While we assessed program participation at the level of the participant, limited or excessive program availability could have altered identified outcomes.

## Conclusion

Our study reveals real-world disparities among support group/service participation for individuals diagnosed with or indirectly affected by cancer within a Midwest, urban demographic. Like previous reports, we found increased rates of utilization among younger individuals, females, those of white race, of higher SES, and having a breast cancer diagnosis. We found that participant age decreased when transitioning to include virtual program delivery, with increased participation among more passive programs and decreased participation among programs benefitting from interpersonal connection and camaraderie. In a society that increasingly relies on virtual communication and interaction, we should not forget the importance of community development and the gravity of in-person connection among those diagnosed with or impacted by cancer.

## Data Availability

No datasets were generated or analysed during the current study.
